# The relationship between dietary inflammation potential, dietary oxidative balance score, and female reproductive function: a mediation analysis of obesity indicators

**DOI:** 10.3389/fendo.2025.1517318

**Published:** 2025-05-19

**Authors:** Mingyue Liang, Xuanhui Wu, Ying Xie, Ying Wang, Bin Luo, Hongmei Xue, Zengning Li

**Affiliations:** ^1^ School of Public Health, Hebei Medical University, Shijiazhuang, China; ^2^ Department of Clinical Nutrition, The First Hospital of Hebei Medical University, Shijiazhuang, China; ^3^ Hebei Key Laboratory of Nutrition and Health, Shijiazhuang, China; ^4^ School of Public Health, Hebei University, Baoding, China

**Keywords:** dietary inflammation, infertility, oxidative stress, national health and nutrition examination survey (NHANES), mediation analysis

## Abstract

**Background:**

Dietary inflammation and oxidative stress have been linked to reproductive health, and weight gain has also been found to play an important role in female reproductive function specifically. In this study we explore the effects of dietary inflammatory index (DII) and dietary oxidative balance score (DOBS) on infertility and sex hormones, and clarify the mediating role of obesity on these effects.

**Methods:**

A total of 1,774 subjects from the 2013-2016 National Health and Nutrition Examination Survey (NHANES) were selected. The DII and DOBS were determined by aggregating data on 26 and 17 dietary components obtained by dietary recall, respectively. Infertility (yes or no, self-reported in questionnaire) and serum gonadal hormones including total testosterone (TT), estradiol (E_2_), and sex hormone-binding globulin (SHBG) were considered as main outcome variables. Multivariate logistic regression and restricted cubic splines (RCS) were applied for further analysis, and mediation models were constructed to figure out the mediating role of obesity indicators.

**Results:**

The prevalence of infertility among American women of childbearing age was 12.66%. There were significant linear relationships between the DII and DOBS, and infertility and serum SHBG (*p* for overall < 0.05). Consuming foods with higher DII was significantly associated with higher risk of infertility (OR: 1.86; 95% CI: 1.20-2.89) and lower levels of SHBG (β: -9.98; 95% CI: -19.45–0.51). Compared to the lowest DOBS category, the adjusted beta estimates for SHBG and E_2_ were 12.03 (95% CI: 2.01-22.04) and 134.58 (95% CI: 3.46-266.24) in the highest DOBS group. However, anti-inflammatory and anti-oxidative diets reduced the risk of infertility by 51% and increased SHBG levels by 14.54 nmol/L. Interestingly, obesity indicators mediated the associations of dietary inflammation and oxidative stress potential with infertility and SHBG.

**Conclusions:**

Dietary inflammation and oxidative stress are strongly associated with the risk of infertility and serum SHBG levels, indicating that anti-inflammatory and anti-oxidative diets may mitigate fertility disorders that result from obesity.

## 1 Introduction

Infertility is a disease of the male or female reproductive system defined by the failure to achieve a pregnancy after 12 months or more of regular unprotected sexual intercourse and is a major health challenge globally (1). According to a World Health Organization (WHO) report published in 2023, the lifetime prevalence of adult infertility worldwide is approximately 17.5% (1). The prevalence of infertility among married women aged 15-44 in the U.S. rose from 6.7% in 2011-2015 to 8.7% in 2015-2019, according to the National Center for Health Statistics (2). Polycystic ovary syndrome (PCOS) is the most common cause of anovulatory infertility, and sex steroid hormones and related binding proteins have been linked to its prevention and diagnosis (3–5). Inflammation and oxidative stress play important roles in reproduction generally speaking as well. Several studies have demonstrated that pro-inflammatory cytokines and reactive oxygen species alter estrous cyclicity, steroidogenesis, and ovulation, increasing the risk of infertility (6, 7), and diet also has the potential to regulate inflammation and oxidative stress (8).

Dietary inflammatory index (DII) and dietary oxidative balance score (DOBS), calculated based on the inflammatory and oxidative effects of dietary nutrients, are ideal indicators for assessing the effects of diet on inflammation and oxidative stress (9, 10) and have been shown to predict pertinent biomarkers for biological aging (11). In female adolescents in particular, strong associations between DII with SHBG and TT have been reported (12). Moreover, a longitudinal study revealed that pro-inflammatory diets can put women at risk for infertility (13). Nevertheless, the majority of prior studies focused on the individual effects of dietary anti-inflammatory potential. The combined effects of dietary inflammation and oxidative stress on reproductive health therefore warrant further investigation.

Obesity has likewise been shown to be an important factor in women’s reproductive health. Previous studies have found that insulin resistance and abnormal lipid metabolism caused by being overweight and obese can affect the secretion of female reproductive hormones, lead to abnormal ovulation, and increase the incidence of female infertility (14, 15). Whether a weight-loss diet can regulate sex hormone levels and improve female infertility is also attention question worth answering. Based on the above relationship between diet, obesity, and reproductive function, one may naturally assume that the relationships between dietary inflammatory and oxidative potential and female reproductive function may be mediated by obesity.

In order to elucidate the combined effects of dietary anti-inflammatory and anti-oxidative potential on female reproductive function, we analyzed the relationships between DII, DOBS, DII&DOBS and reproductive function indicators including infertility, SHBG, E_2_, and T. Further, obesity indicators were included into the models as mediating variables. The data were sourced from the NHANES cycles of 2013-2016.

## 2 Materials and methods

### 2.1 Study population

The NHANES is a complex, stratified, and multistage study conducted by the National Center for Health Statistics (NCHS) in an effort to understand the nutritional and health status of Americans. Detailed information about NHANES is publicly available at https://www.cdc.gov/nchs/nhanes/index.htm. Prior to the survey, all protocols received approval from the Institutional Review Board of the NCHS, and informed consent was obtained from all participants.

**Figure 1:**
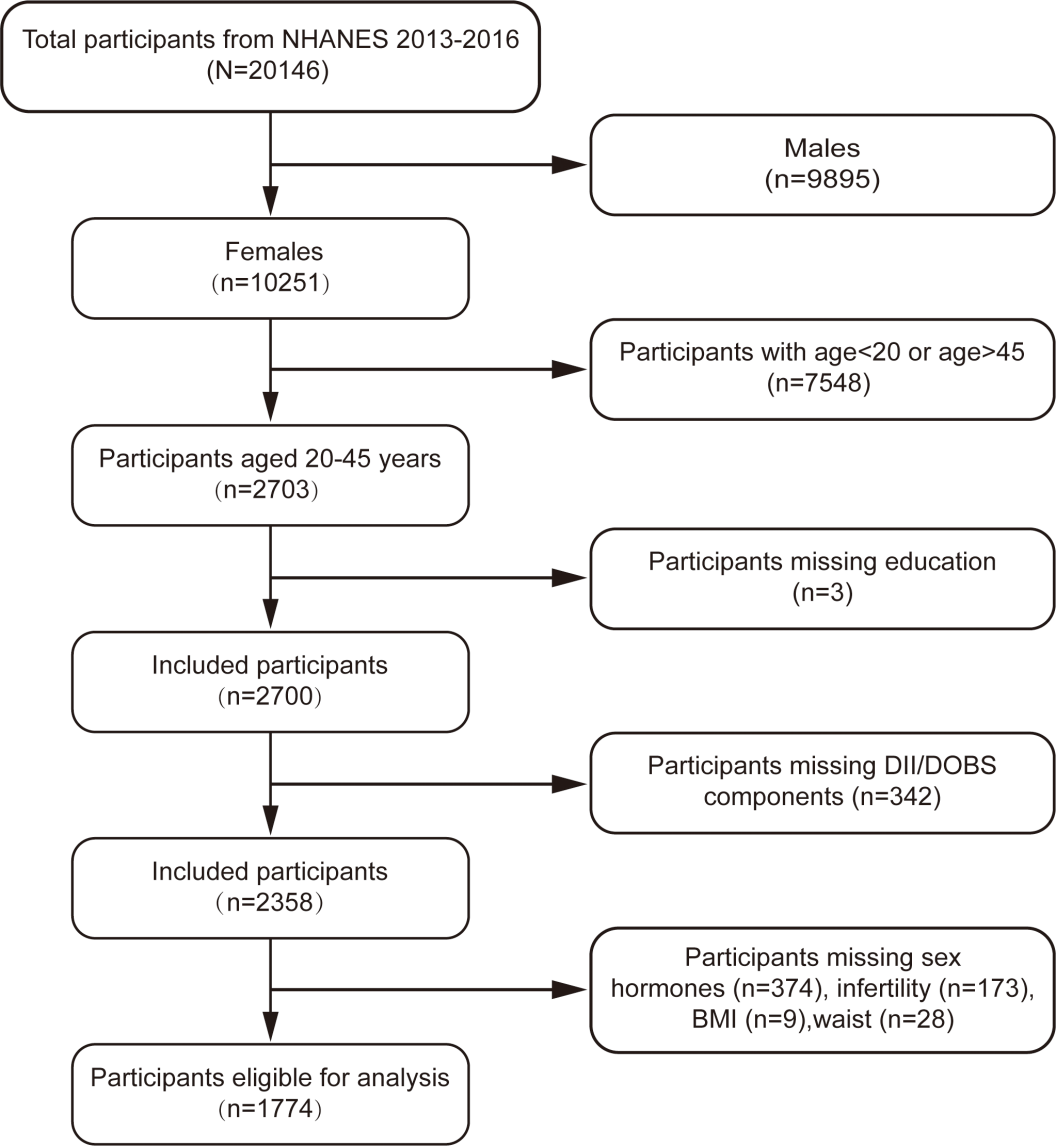
Flowchart depicting participant selection.

This cross-sectional study was based on the 2013-2016 cycle of the NHANES and included women aged 20-45 years. Individuals fulfilling any of the following criteria were excluded: (a) males (n=9,895); (b) age< 20 or age> 45 (n=7,548); (c) missing data for DII/DOBS components (n=342); or (d) missing outcomes or mediating variables (n=584). After applying these filters a total of 1,774 participants were included in the final analysis (**Figure 1**).

### 2.2 Assessment of DII

In the NHANES, dietary nutrient intake was assessed by a 24-hour dietary review. Participants were asked about the types and amounts of food they had consumed by professional technicians. The intake of each food component was then estimated using the University of Texas Food Intake Analysis System and the USDA Survey Nutrient Database (16).

The DII has been described in detail in previous studies (10) and is based on literature review and population data. The score considered 45 dietary components associated with dietary inflammatory potential and their representative dietary intake ranges. In this study, 26 of the above 45 dietary components were selected for the DII calculation. Previous studies have shown that the predictive power of DII remains stable when it is calculated using fewer than 30 dietary components (17). Details of the dietary components used to calculate the DII can be found in **Supplementary Table S1**. First, the Z-score of a certain dietary component was calculated as follows: (daily intake - global daily mean intake)/the standard deviation * the overall inflammatory effect score. Second, this value was converted to a percentile. To achieve a symmetrical distribution with values centered on 0 and bounded between -1 (maximally anti-inflammatory) and 1 (maximally pro-inflammatory), each percentile score was doubled and then 1 was subtracted from the result. Finally, the DIIs of the 26 individual dietary components were added together to obtain the participant’s DII. Higher DIIs indicate greater dietary inflammatory potential.

### 2.3 Assessment of DOBS

The DOBS was calculated by combining predetermined scores of pro- and anti-oxidative factors from the diet, considering a total of 17 nutrients, including 3 pro-oxidants and 14 anti-oxidants. These nutrients have been well-established in previous studies for their role in determining the DOBS (11, 18). Anti-oxidants were scored from 1 to 3, and pro-oxidants were scored inversely after categorizing the dietary variable into tertiles. Alcohol intake was categorized a nondrinker, nonheavy drinker, and heavy drinker, with scores of 3, 2, and 1, respectively. Additionally, vitamin C, beta-carotene, and vitamin B12 were log-transformed for better distribution approximation. Detailed information on the scoring system for DOBS components can be found in **Supplementary Table S2**. The participant’s DOBSs were calculated as the sum of scores for each dietary ingredient selected.

We also set DII&DOBS as a variable to evaluate the combined effect of the two indicators in order to study the potential effects of diets with pro-inflammatory and pro-oxidative properties versus diets with anti-inflammatory and anti-oxidative proper-ties on female reproductive function (11, 18). Participants were divided into three groups based on their DII and DOBS values: the pro-inflammatory and pro-oxidative diet group (those in the third tertile of DII and the first tertile of DOBS), the anti-inflammatory and anti-oxidative diet group (those in the first tertile of DII and the third tertile of DOBS), and the composite diet group (those who did not fall in either of the above two groups).

### 2.4 Assessment of serum sex hormones, SHBG and infertility

The outcome variable of infertility was derived from each woman’s self-report on the Reproductive Health Questionnaire (RHQ074). When asked “Have you ever attempted to become pregnant over at least a year without becoming pregnant?” Women who responded “yes” were labeled as “ever-infertile” and those who responded “no” were labeled as “fertile” (19).

Blood samples were drawn from all study participants’ antecubital veins by a trained phlebotomist, and serum specimens were stored under -30°C before being shipped to the National Center for Environmental Health for testing. Serum TT and E_2_ were measured using isotope dilution liquid chromatography tandem mass spectrometry (ID-LC-MS/MS), and SHBG was quantified based on the reaction of SHBG with immuno-antibodies and chemo-luminescence measurements of the reaction products via a photomultiplier tube. Details of the analytical methodology can be found in the laboratory procedure manual on the NHANES website (20, 21).

### 2.5 Assessment of BMI, WC, and covariates

The mediating variables considered in this study were BMI and waist circumference (WC). WC was measured at the iliac crest by a tape measure to the nearest millimeter (22), and BMI was calculated as weight in kilograms divided by the square of height in meters. According to previous research, individuals with a BMI≥30.0 kg/m^2^ were classified as “obese” and the rest as “nonobese”, and women with a WC≥ 88 cm were labeled as “high-WC” (23).

The confounders adjusted in this study included age (years), race (nonHispanic White/nonHispanic Black/Mexican American/other race/ethnicity), education level (below high school/high school degree/some college or AA degree/college graduate or above), marital status (married or living with partner/live alone), family income to poverty ratio (PIR) (< 1/≥ 1/miss), smoking status (never smoker/former smoker/current smoker), physical activity (≥ 600MET/< 600MET), total energy intake (kcal), total cholesterol (mmol/L), triglyceride (mmol/L), LDL-cholesterol (mmol/L), HDL-cholesterol (mmol/L) and insulin resistance index (HOMA-IR). HOMA-IR = fasting insulin (mU/L) * fasting glucose (mmol/L)/22.5 (24).

### 2.6 Statistical analysis

Individual sample weights were established based on the NHANES recommended sample weight on dietary day-one (WTDRD1) records. During a baseline characteristic analysis, continuous variables were expressed as weighted means (standard errors), and categorical variables were expressed as sample numbers (weighted percentages). To examine differences in the characteristics of variables between the DII, DOBS, and different combinations of DII and DOBS values, univariate analysis was performed using ANOVA for differences in weighted means of continuous variables and the chi-squared test was carried out for differences in weighted percentages of categorical variables in order to characterize the total population. Moreover, to study the effects of obesity, we stratified the data by obesity and high-WC, and further analyzed the characteristics of DII, DOBS, and DII&DOBS in infertile and fertile groups.

The continuous variables SHBG, TT, and E_2_ were transformed into binary variables according to their relation to the median. Binomial logistic regression models were then used to investigate the associations between DII, DOBS, and different combinations of DII and DOBS with infertility and sex hormones. Subsequently, the best fit dose-response curves for the correlations between DII and DOBS with indicators of reproductive function were shaped by restricted cubic spline (RCS) regression. Multivariate linear regression models were also performed to assess relationships between DII, DOBS, and different combinations of DII and DOBS with continuous indicators of reproductive function (SHBG, TT, and E_2_) and traditional obesity (BMI and WC). All of the above regression models were adjusted for age, race, education, marital status, PIR, smoking status, physical activity and total energy intake.

Mediation analysis was used to identify the mediation effects of obesity indicators on the relationships between dietary inflammation and oxidative stress potential and female reproductive function. All statistical analysis was conducted with R 4.3.3, and a two-sided *p* value < 0.05 was assumed to indicate a statistically significant test result for all tests.

## 3 Results

### 3.1 Baseline characteristics

Baseline characteristics of individuals grouped by DII tertile, DOBS tertile, and different combinations of DII and DOBS are shown in **Supplementary Table S3**. The mean age of subjects was 32.53 ± 0.30 years. Of the participants, 55.71% were non-Hispanic white, and the weighted prevalence of infertility was 12.66%. With the exception of physical activity and obesity, there were significant differences in study variables between different DII/DOBS tertiles (*p*< 0.05). Participants consuming pro-inflammatory and pro-oxidative diets were more likely to be Hispanic white, to have lower education, to have lower wealth, to have less physical activity, to have higher BMI and WC, to have lower total energy intake, to live alone, and to smoke (*p*< 0.05).

In the stratified analysis, significant differences in DII and DOBS between the infertile and fertile groups were only observed in women with obesity and high-WC (*p*< 0.05), suggesting that obesity indicators may be crucial factors (**Table 1**).

**Table 1:** Characteristics of DII, DOBS, and DII&DOBS in fertile and infertile groups stratified by obesity indicators^a^.

Dietary type	Non-obese (n=1046)			Obese^c^ (n=728)		
	Fertile	Infertile	*p* ^d^	Fertile	Infertile	*p*
**DII** ^b^	1.04 (0.10)	1.13 (0.21)	0.68	1.35 (0.12)	1.81 (0.14)	0.018
**DOBS** ^b^	34.61 (0.35)	35.09 (0.67)	0.50	34.08 (0.45)	32.42 (0.53)	0.018
**DII&DOBS (%)**			0.24			0.11
Pro-inflammatory and pro-oxidative diet	229 (22.74)	22 (20.13)		201 (29.96)	42 (37.89)	
Anti-inflammatory and anti-oxidative diet	246 (26.62)	21 (20.99)		142 (23.52)	21 (15.51)	
Composite diet	479 (50.64)	49 (58.88)		264 (46.52)	58 (46.60)	

Dietary type	Normal-WC^b^ (n=671)			High-WC^c^ (n=1103)		
	Fertile	Infertile	*p*	Fertile	Infertile	*p*
**DII**	0.94 (0.11)	0.94 (0.11)	0.63	1.31 (0.10)	1.64 (0.14)	0.038
**DOBS**	34.84 (0.38)	34.78 (1.02)	0.96	34.10 (0.34)	33.19 (0.50)	0.11
**DII&DOBS (%)**			0.45			0.16
Pro-inflammatory and pro-oxidative diet	132 (20.78)	14 (23.49)		298 (28.69)	50 (32.30)	
Anti-inflammatory and anti-oxidative diet	169 (28.20)	12 (20.71)		219 (23.53)	30 (16.98)	
Composite diet	314 (51.02)	30 (55.80)		429 (47.78)	77 (50.72)	

a Values are presented as weighted mean (SE) for continuous variables, and n (%) for categorical variables.

b DII, dietary inflammatory index; DOBS, dietary oxidative balance score; WC, waist circumference.

c Individuals with a BMI ≥30.0 kg/m^2^ were classified as “obese”, and women with a WC ≥ 88 cm were labeled as “high-WC” (23).

d Two independent sample *t*-test was used for differences in weighted means of continuous variables, and the chi-squared test was used for differences in weighted percentages of categorical variables.

### 3.2 Multiple logistic regression analysis

**Figure 2:**
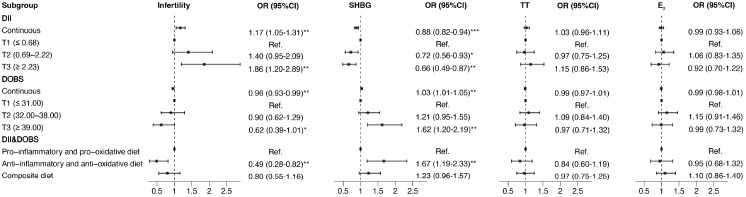
Forest plot of the associations of DII, DOBS, and DII&DOBS with indicators of reproductive function in binomial logistic regression models. SHBG, TT, and E^2^ were transformed into binary variables by comparing them to their respective medians. Models were adjusted for age, race, education, marital status, PIR, smoking status, physical activity, and total energy intake. *p<0.05, **p<0.01, ***p<0.001. T, tertile; SHBG, sex hormone-binding globulin; TT, total testosterone; E_2_, estradiol; PIR, family income-to-poverty ratio.

**Figure 2** shows the relationship between DII, DOBS, DII&DOBS and infertility and sex hormones as determined by binomial logistic regression. In the model that adjusts for all confounding, the highest DII tertile was associated with increased risk of infertility and low-SHBG. For DOBS, participants in T3 had significantly lower risk of infertility (OR: 0.62, 95% CI 0.39-1.01) and higher SHBG levels (OR: 1.62, 95% CI 1.20-2.19) compared to those in T1. Additionally, women who consumed anti-inflammatory and anti-oxidative diets had lower risk of infertility (OR: 0.49, 95% CI 0.28-0.82) and higher SHBG levels (OR: 1.67, 95% CI 1.19-2.33). After further adjusting the current model for total cholesterol, triglyceride, LDL-cholesterol, HDL-cholesterol and HOMA-IR, the association between anti-inflammatory and anti-oxidative diets and infertility remained significant (OR: 0.43, 95% CI 0.18-0.95). For obesity indicators, when DII increased by one unit, the risks of obesity and high-WC increased by 1.15 and 1.12 times, respectively. Furthermore, an anti-inflammatory and anti-oxidative diet was a protective factor for obesity (OR: 0.62, 95% CI 0.44-0.87) and high-WC (OR: 0.63, 95% CI 0.44-0.91) (**Supplementary Figure S1**).

### 3.3 RCS plots of correlations between DII/DOBS and reproductive function indicators

**Figure 3:**
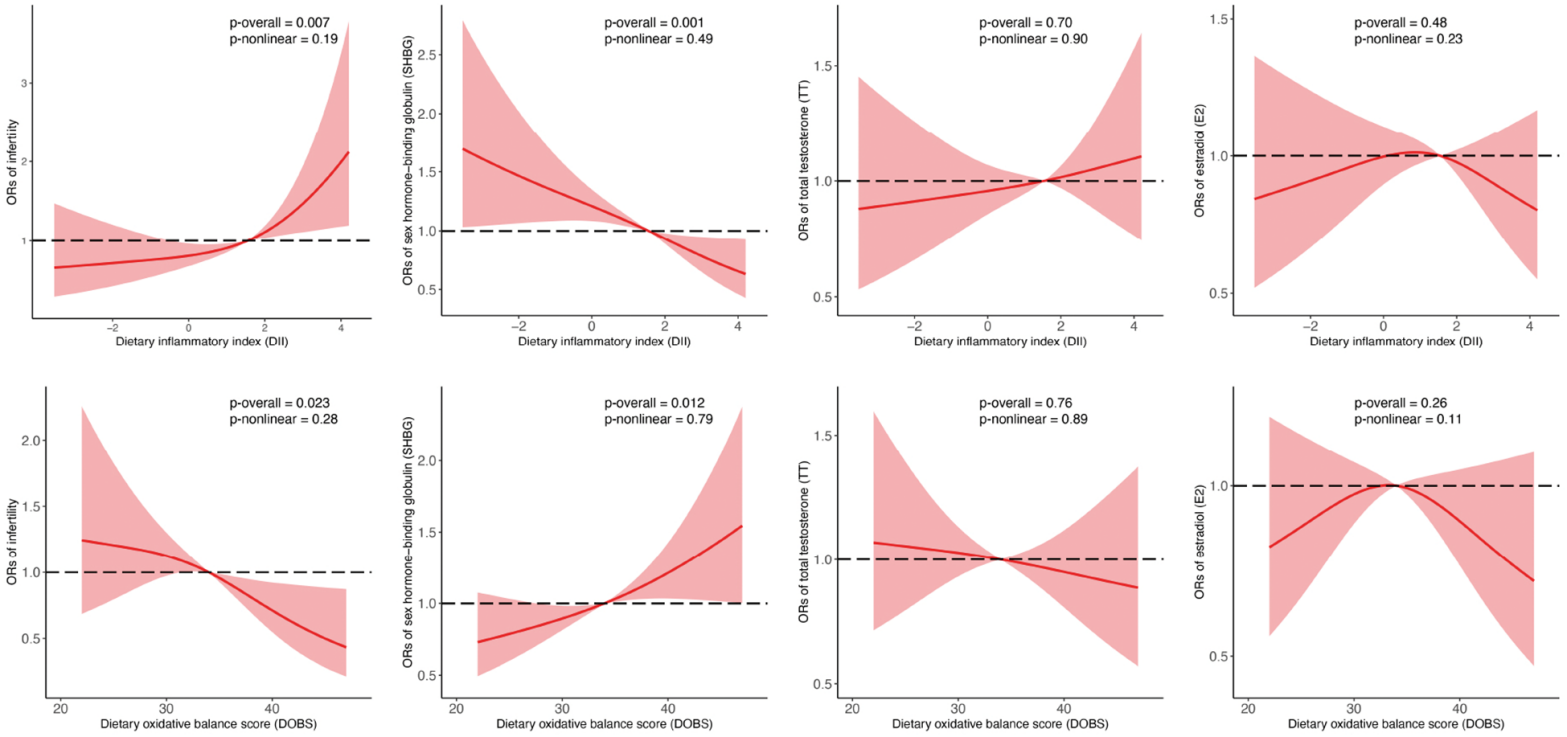
Associations between DII, DOBS, and reproductive function indicators analyzed by binomial logistic regression and RCS. SHBG, TT, and E_2_ were transformed into binary variables by comparing them to their respective medians. Models were adjusted for age, race, education, marital status, PIR, smoking status, physical activity, and total energy intake. SHBG, sex hormone-binding globulin; TT, total testosterone; E_2_, estradiol; PIR, family income-to-poverty ratio. Central estimates are represented by solid red lines, and shaded areas indicate 95% CIs.

In RCS regression, after adjusting for different covariates, we detected significant linear relationships between continuous DII/DOBS and infertility risk and dichotomous SHBG (*p* for overall <0.05, *p* for nonlinear >0.05, **Figure 3**).

### 3.4 Multivariate linear regression analysis

**Figure 4:**
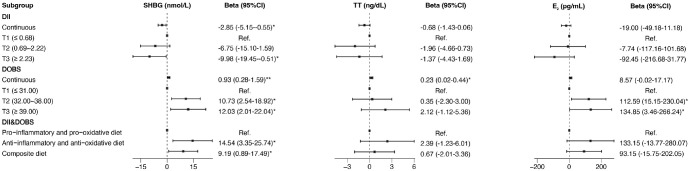
Forest plot of the associations of DII, DOBS, and DII&DOBS with SHBG and sex hormones in multivariate linear regression models. Models were adjusted for age, race, education, marital status, PIR, smoking status, physical activity, and total energy intake. *p<0.05, **p<0.01. T, tertile; SHBG, sex hormone-binding globulin; TT, total testosterone; E_2_, estradiol; PIR, family income-to-poverty ratio.

In the multivariate linear regression models that adjusted for all confounding, there was a strong negative correlation of DII with SHBG (β=-2.85, 95% CI: -5.15–0.55) and strong positive correlations of DII with BMI (β=0.52, 95% CI: 0.27-0.77) and WC (β=1.18, 95% CI: 0.61-1.75). DOBS was positively associated with SHBG (β=0.93, 95% CI: 0.28-1.59) and TT (β= 0.23, 95% CI: 0.02-0.44) and was inversely correlated with BMI (β=-0.08, 95% CI: -0.15–0.01), WC (β=-0.16, 95% CI: -0.33–0.01). An anti-inflammatory and anti-oxidative diet was positively correlated with serum SHBG and negatively correlated with BMI and WC, and the beta estimates (95% CIs) were 14.54 (3.35 to 25.74), -1.67 (-2.90 to -0.44), and -4.15 (-6.92 to -1.38). The results of the multivariate linear regression analysis are shown in **Figure 4** and **Supplementary Figure S2**.

### 3.5 Associations between obesity indicators and reproductive function

**Figure 5:**
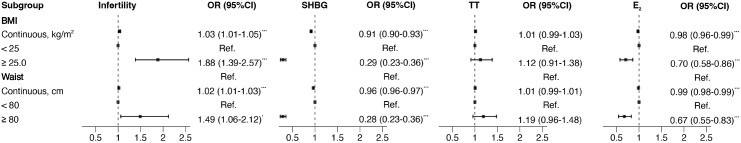
Forest plot of the associations of BMI and WC with indicators of reproductive function in binomial logistic regression models. SHBG, TT, and E_2_ were transformed into binary variables by comparing them to their respective medians. Models were adjusted for age, race, education, marital status, PIR, smoking status, physical activity, and total energy intake. *p<0.05, ***p<0.001. SHBG, sex hormone-binding globulin; TT, total testosterone; E_2_, estradiol; BMI, body mass index; WC, waist circumference; PIR, family income-to-poverty ratio.

Multivariate linear regression and logistic regression models were developed to evaluate the associations between obesity indicators and infertility risk and sex hormone levels. BMI (OR=1.03, 95% CI: 1.01-1.05) and WC (OR=1.02, 95% CI: 1.01-1.03) were strongly related to a higher risk of infertility after adjusting for confounders (**Figure 5**). More specifically, BMI was negatively associated with SHBG (β=-1.53, 95% CI: -1.95–1.11) and positively associated with TT (β=0.14, 95% CI: 0.01-0.27). The beta estimates (95% CI) between WC and SHBG, TT, and E_2_ were -0.59 (-0.78 to -0.40), 0.06 (0.01 to 0.12) and 2.58 (0.10 to 5.05), respectively, after adjusting for covariates (**Figure 5**, **Supplementary Figure S3**).

### 3.6 Mediating effect analysis

**Figure 6:**
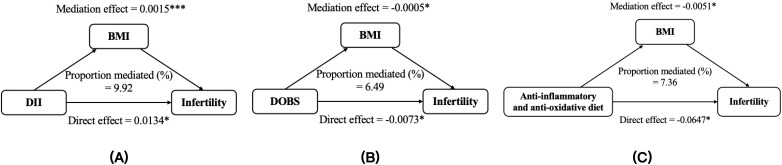
BMI mediated the relationships between DII **(A)**, DOBS **(B)**, anti-inflammatory and anti-oxidative diet **(C)** and infertility. Models were adjusted for age, race, education, marital status, PIR, smoking status, physical activity, total energy intake. * *p* < 0.05, *** *p* < 0.001. DII, dietary inflammatory index; DOBS, dietary oxidative balance score; BMI, body mass index.

**Figure 7:**
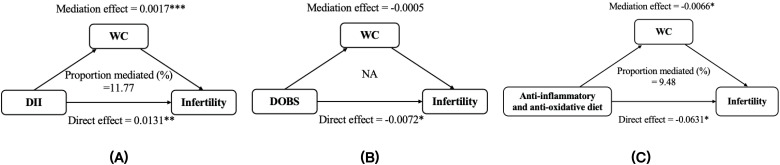
The mediation analysis of WC on the relationships between DII **(A)**, DOBS **(B)**, anti-inflammatory and anti-oxidative diet **(C)** and infertility. Models were adjusted for age, race, education, marital status, PIR, smoking status, physical activity, total energy intake. * *p* < 0.05, ** *p* < 0.01, *** *p* < 0.001. DII, dietary inflammatory index; DOBS, dietary oxidative balance score; WC, waist circumference

Mediation statistical models were performed to ascertain whether BMI and WC had mediation effects on any of the above associations. The proportions of the indirect effects on DII, DOBS, anti-inflammatory and anti-oxidative diet, and infertility mediated by BMI were measured at 9.92, 6.49, and 7.36, respectively. Similarly, the percentages of the indirect influences on DII, anti-inflammatory and anti-oxidative diet, and infertility mediated by WC were determined to be 11.77 and 9.48, respectively (**Figures 6**, **7**). Except for the effect of DOBS on SHGB, BMI and WC also significantly mediated the associations of dietary inflammation and oxidative stress scores with SHBG (**Supplementary Figures S4**, **S5**).

## 4 Discussion

This study demonstrates that the dietary potential for inflammation and oxidative stress, as indicated by scores derived from pro-inflammatory and anti-oxidative dietary components in an individual’s diet, are linked to an increased risk of infertility and lower levels of SHBG. Additionally, the significant mediating effect of BMI and WC suggests that anti-inflammatory and/or anti-oxidant diets for weight loss may be an effective way to prevent infertility and low SHBG levels.

Previous studies have shown that consumption of high DII diets is associated with increased risk of chronic inflammation activation, obesity, PCOS, and infertility (25–27). DOBS is associated with rheumatoid arthritis, biological aging, and all-cause and disease-specific mortality (11, 28, 29). Teng and colleagues built a nomogram model based on anti-oxidative intake (vitamin C, vitamin E, selenium) and showed significant predictive capacity for stroke risk with an area under the curve (AUC) of 77.4% (76.3%-78.5%) (30). However, there may be a synergistic effect between dietary inflammation and oxidative stress components, and there are currently few articles that consider their joint effects. In this paper, we found that an anti-inflammatory and anti-oxidative diet may be a protective factor for female reproductive function. Compared to a pro-inflammatory pro-oxidative diet, an anti-inflammatory anti-oxidative diet reduced the risk of infertility by 51% and increased levels of SHBG by 14.54 nmol/L. Decreased SHBG levels increase the bioavailability of androgens, which in turn leads to progression of ovarian pathology, anovulation, and the phenotypic characteristics of PCOS. SHBG has been found to be an early biomarker and therapeutic target for PCOS (4). From a public health perspective, our findings suggest that an anti-inflammatory and anti-oxidative diet is a potential independent factor affecting reproductive health. Promoting such a diet may reduce the risk of infertility in the population.

Inflammation and oxidative stress in the body are influenced by dietary and lifestyle factors (31–33). One previous study has suggested that anti-inflammatory and anti-oxidative components in the diet may affect the balance of inflammation and oxidative stress (11), and positive associations have observed between DII/DOBS values and levels of CRP, IL6, TNF-α in the blood (34). Previous studies have shown that chronic inflammatory activation and oxidative stress may contribute to infertility as well (33). Oxidative stress can inhibit the expression and secretion of SHBG by down-regulating HNF-4α, which may be an important factor promoting the occurrence of hyperandrogenemia in PCOS (35).

Our study reveals the associations between dietary inflammation and oxidative stress potential and obesity. In mediating effect analysis, the significant mediation effect indicating that obesity indicators, specifically BMI and WC, serve as important pathways through which anti-inflammatory and anti-oxidative diets influence infertility. This finding underscores the importance of dietary interventions aimed at obese women, emphasizing the need to prioritize anti-inflammatory and antioxidant components to enhance reproductive health. In addition, the significant direct effect suggesting that there are other potential mechanisms besides obesity. For instance, pro-inflammatory and pro-oxidative diets such as unhealthy hypercaloric diets, excessive consumption of saturated and trans-fatty acids, and processed foods can lead to insulin resistance and diabetes, which are major causes of increased risk of infertility and hormonal disorders in women (36, 37). It has also been confirmed in mice that were fed a high-fat diet that the mRNA expression levels of oxidative stress markers (such as ROS) in oocytes were significantly increased, and the levels of anti-oxidative markers (such as GSH-Px, SOD, and CAT) were decreased. Oxidative stress was activated, which damaged mitochondrial function, led to oocyte apoptosis, and ultimately reduced oocyte quality (38).

In fact, many studies have demonstrated a causal relationship between obesity and impaired reproductive function. For example, a Mendelian randomization study showed that BMI, waist-to-hip ratio (WHR), and WHR adjusted for BMI may cause PCOS (39). Compared to age-matched controls, obese women had a higher risk of ovulatory sub-fertility and anovulatory infertility (40–42). Although anovulation can be overcome with ovarian stimulation, obese women have decreased responsiveness to gonadotropins, decreased oocyte retrieval, decreased oocyte quality, reduced rates of pre-implantation embryo development, and increased risk for miscarriage compared to their lean counterparts (15, 40, 43). Hunter et al. conducted a meta-analysis of randomized controlled trials (RCTs) on the effectiveness of weight loss interventions in improving obesity and infertility outcomes and found that women randomized to a combination of diet and exercise intervention were more likely to become pregnant, risk ratio (RR) = 1.87 (95% CI 1.20, 2.93) and achieve a live birth RR = 2.20 (95% CI 1.23, 3.94) (44). Furthermore, several studies using rodent models have shown that obesity has a negative impact on ovarian function. For example, rats fed a high-fat diet gained weight and had irregular estrus cycles, manifested by longer estrous intervals and shorter estrus periods (45, 46). Additionally, in obese mice and rats, increased ovarian granulosa cell apoptosis induces follicular atresia, which reduces egg number, egg quality, and ovaria-related hormone synthesis (47). All of these research results suggest that the impact of obesity on female reproductive function cannot be ignored.

In the univariate analysis stratified by obesity indicators, significant differences in DII and DOBS were only found in women with obesity or high-WC. Additionally, obesity indicators mediated the association between anti-inflammatory and anti-oxidative diets and infertility (BMI: 7.36% and WC: 9.48%). Our results specifically indicate that significant anti-inflammatory and anti-oxidative diets that reduce BMI and WC may be an effective way to prevent and treat infertility. Beyond that, compared with only considering a single indicator, stronger association were observed between anti-inflammatory and anti-oxidative diets and infertility, indicating a possible combined effect of DII and DOBS. In clinical interventions, particularly for obese women, we recommend the consumption of foods rich in antioxidants and anti-inflammatory properties, such as fresh fruits and vegetables, fruits, vegetables, legumes and dietary unsaturated fats (48, 49). Additionally, it is advisable to reduce the intake of high-fat, sugar, ultra-processed foods, red meat (50, 51).

The present study has several notable strengths. First, the NHANES was based on a large sample population and a complex sampling design, and the infertility rate was nationally representative. Second, this study explored the linear and nonlinear associations between dietary inflammation index and oxidative balance score with infertility and reproductive hormones, and further examined the potential mediating role of obesity, thereby providing a treatment possibility for obesity-related reproductive dysfunction.

However, the study also has some limitations. First, due to the cross-sectional study design, a causal association cannot be established and needs to be explored in future, longitudinal studies. Second, the 24-hour dietary recall is dependent on respondents’ memories, making it potentially subject to recall bias. Thirdly, the generalizability of our findings needs to be further validated in populations outside of NHANES. Fourth, in addition to WC and BMI, indicators such as percentage of body fat and visceral fat area can be included in future studies to reflect obesity more comprehensively.

## Data Availability

The raw data supporting the conclusions of this article will be made available by the authors, without undue reservation.
