# Supplementary material for: The relationship between dietary inflammation potential, dietary oxidative balance score, and female reproductive function: a mediation analysis of obesity indicators
Source: Front Endocrinol (Lausanne). 2025 May 19;16:1517318. doi: 10.3389/fendo.2025.1517318 (PMC12127156; doi:10.3389/fendo.2025.1517318)
Supplement: Supplementary file 1 [file DataSheet1.docx]

Supplementary Material

**Supplementary Table 1.** Dietary composition included in the DII calculation

| Dietary composition  parameter | Raw inflammatory effect score | Overall inflammatory effect score | Global daily mean intake (units/d) | SD |
| --- | --- | --- | --- | --- |
| Alcohol (g) | -0.278 | -0.278 | 13.98 | 3.72 |
| Vitamin B_12_ (μg) | 0.205 | 0.106 | 5.15 | 2.7 |
| Vitamin B_6_ (mg) | -0.379 | -0.365 | 1.47 | 0.74 |
| β-Carotene (μg) | -0.584 | -0.584 | 3718 | 1720 |
| Caffeine (g) | -0.124 | -0.110 | 8.05 | 6.67 |
| Carbohydrate (g) | 0.109 | 0.097 | 272.2 | 40.0 |
| Cholesterol (mg) | 0.347 | 0.110 | 279.4 | 51.2 |
| Energy (kcal) | 0.180 | 0.180 | 2056 | 338 |
| Total fat (g) | 0.298 | 0.298 | 71.4 | 19.4 |
| Fibre (g) | -0.663 | -0.663 | 18.8 | 4.9 |
| Folic acid (μg) | -0.207 | -0.190 | 273.0 | 70.7 |
| Fe (mg) | 0.032 | 0.032 | 13.35 | 3.71 |
| Mg (mg) | -0.484 | -0.484 | 310.1 | 139.4 |
| MUFA (g) | -0.019 | -0.009 | 27.0 | 6.1 |
| Niacin (mg) | -1.000 | -0.246 | 25.90 | 11.77 |
| Protein (g) | 0.049 | 0.021 | 79.4 | 13.9 |
| PUFA (g) | -0.337 | -0.337 | 13.88 | 3.76 |
| Riboflavin (mg) | -0.727 | -0.068 | 1.70 | 0.79 |
| Saturated fat (g) | 0.429 | 0.373 | 28.6 | 8.0 |
| Se (μg) | -0.191 | -0.191 | 67.0 | 25.1 |
| Thiamin (mg) | -0.354 | -0.098 | 1.70 | 0.66 |
| Vitamin A (RE) | -0.401 | -0.401 | 983.9 | 518.6 |
| Vitamin C (mg) | -0.424 | -0.424 | 118.2 | 43.46 |
| Vitamin D (μg) | -0.446 | -0.446 | 6.26 | 2.21 |
| Vitamin E (mg) | -0.419 | -0.419 | 8.73 | 1.49 |
| Zn (mg) | -0.313 | -0.313 | 9.84 | 2.19 |

**Supplementary Table 2.** Scoring Principles of DOBS

| **Dietary component** | **Property** | **DOBS score** | | |
| --- | --- | --- | --- | --- |
|  |  | **1** | **2** | **3** |
| Dietary fiber (g/d) | Antioxidant | ≤11.60 | 11.61-18.00 | ≥18.01 |
| Ln-transformed carotene (μg/d) | Antioxidant | ≤5.89 | 5.90-7.22 | ≥7.23 |
| Ln-transformed vitamin B12 (μg/d) | Antioxidant | ≤0.908 | 0.909-1.613 | ≥1.614 |
| Ln-transformed vitamin C (mg/d) | Antioxidant | ≤3.384 | 3.385-4.533 | ≥4.534 |
| Vitamin B2 (mg/d) | Antioxidant | ≤1.28 | 1.29-2.09 | ≥2.10 |
| Niacin (mg/d) | Antioxidant | ≤16.58 | 16.59-25.15 | ≥25.16 |
| Vitamin B6 (mg/d) | Antioxidant | ≤1.24 | 1.25-2.11 | ≥2.12 |
| Folic acid (μg/d) | Antioxidant | ≤78 | 79-171 | ≥172 |
| Vitamin E (mg/d) | Antioxidant | ≤5.80 | 5.81-10.02 | ≥10.03 |
| Calcium (mg/d) | Antioxidant | ≤602 | 603-983 | ≥984 |
| Magnesium (mg/d) | Antioxidant | ≤202 | 203-298 | ≥299 |
| Zinc (mg/d) | Antioxidant | ≤7.58 | 7.59-11.33 | ≥11.34 |
| Copper (mg/d) | Antioxidant | ≤0.81 | 0.82-1.28 | ≥1.29 |
| Selenium (μg/d) | Antioxidant | ≤77.10 | 77.11-117.20 | ≥117.21 |
| Iron (mg/d) | Prooxidant | ≥14.24 | 9.56-14.23 | ≤9.55 |
| Total fat (gm/d) | Prooxidant | ≥85.38 | 55.06-85.37 | ≤55.05 |
| Alcohol (g/d) | Prooxidant | ≥15.01 | 0.01-15 | 0 |

**Supplementary Table 3.** Weighted characteristics of the study population by DII tertile, DOBS tertile, and DII&DOBS. ^a^

|  | **Total** | **Dietary inflammatory index (DII)** | | | | **Dietary oxidative balance score (DOBS)** | | | | **Different combinations of DII and DOBS** | | | |
| --- | --- | --- | --- | --- | --- | --- | --- | --- | --- | --- | --- | --- | --- |
|  |  | **T1^b^**  **(≤0.68)** | **T2**  **(0.69~2.22)** | **T3**  **(≥2.23)** | ***p*^d^** | **T1**  **(≤29.00)** | **T2**  **(30.00~38.00)** | **T3**  **(≥39.00)** | ***p*** | **Pro-inflammatory and pro-oxidative diet** | **Anti-inflammatory and antioxidative diet** | **Composite diet** | ***p*** |
| **Age, years** | 32.53 (0.30) | 33.40 (0.43) | 32.14 (0.52) | 32.03 (0.40) | 0.009 | 31.96 (0.37) | 32.53 (0.54) | 33.24 (0.37) | 0.05 | 32.00 (0.50) | 33.36 (0.38) | 32.40 (0.47) | 0.05 |
| **Race/ethnicity (%)** |  |  |  |  | ＜0.001 |  |  |  | 0.013 |  |  |  | 0.001 |
| Non-Hispanic White | 612 (55.71) | 196 (56.64) | 216 (58.54) | 200 (51.89) |  | 229 (54.97) | 208 (55.86) | 175 (56.69) |  | 170 (52.89) | 144 (56.93) | 298 (56.73) |  |
| Non-Hispanic Black | 351 (12.86) | 74 (7.86) | 122 (12.86) | 155 (17.67) |  | 171 (16.65) | 103 (10.90) | 77 (9.90) |  | 135 (18.54) | 53 (8.37) | 163 (11.75) |  |
| Mexican American | 333 (13.54) | 135 (15.73) | 108 (12.84) | 90 (11.06) |  | 100 (11.30) | 114 (13.35) | 119 (15.55) |  | 73 (11.06) | 101 (15.63) | 159 (13.24) |  |
| Other | 478 (17.90) | 187 (19.77) | 145 (15.76) | 146 (19.38) |  | 162 (17.08) | 169 (19.89) | 147 (17.86) |  | 116 (17.51) | 132 (19.07) | 230 (18.28) |  |
| **Education (%)** |  |  |  |  | ＜0.001 |  |  |  | ＜0.001 |  |  |  | ＜0.001 |
| Below high school | 294 (12.14) | 95 (10.82) | 82 (8.80) | 117 (17.48) |  | 124 (14.25) | 84 (10.52) | 86 (11.93) |  | 96 (15.55) | 70 (11.24) | 128 (11.08) |  |
| High school | 337 (18.16) | 86 (12.14) | 116 (18.78) | 135 (24.26) |  | 147 (22.42) | 121 (19.86) | 69 (11.00) |  | 115 (24.50) | 58 (11.57) | 164 (18.13) |  |
| Some college or AA degree | 650 (37.39) | 171 (31.48) | 239 (42.81) | 240 (37.83) |  | 281 (44.80) | 200 (32.86) | 169 (33.86) |  | 210 (43.51) | 131 (31.20) | 309 (37.41) |  |
| College graduate or above | 493 (32.31) | 240 (45.56) | 154 (29.61) | 99 (20.43) |  | 110 (18.53) | 189 (36.76) | 194 (43.21) |  | 73 (16.44) | 171 (45.99) | 249 (33.38) |  |
| **Marital status (%)** |  |  |  |  | 0.047 |  |  |  | 0.002 |  |  |  | 0.004 |
| Married or living with partner | 1031 (60.07) | 378 (65.52) | 334 (59.17) | 319 (54.95) |  | 343 (54.36) | 367 (64.38) | 321 (64.58) |  | 256 (53.80) | 273 (66.26) | 502 (61.80) |  |
| Live alone | 743 (39.93) | 214 (34.48) | 257 (40.83) | 272 (45.05) |  | 319 (45.64) | 227 (35.62) | 197 (35.42) |  | 238 (46.20) | 157 (33.74) | 348 (38.20) |  |
| **PIR**^b^ **(%)** |  |  |  |  | ＜0.001 |  |  |  | 0.015 |  |  |  | < 0.001 |
| < 1 | 427 (19.76) | 115 (14.64) | 137 (17.64) | 175 (27.56) |  | 198 (24.42) | 128 (18.76) | 101 (15.22) |  | 150 (27.42) | 85 (14.73) | 192 (18.24) |  |
| ≥ 1 | 1225 (74.32) | 428 (77.66) | 422 (78.05) | 375 (66.71) |  | 416 (69.41) | 429 (75.15) | 380 (79.39) |  | 308 (66.31) | 313 (79.36) | 604 (76.03) |  |
| Miss | 122 (5.92) | 49 (7.70) | 32 (4.31) | 41 (5.73) |  | 48 (6.17) | 37 (6.09) | 37 (5.39) |  | 36 (6.27) | 32 (5.91) | 54 (5.73) |  |
| **Smoking status (%)** |  |  |  |  | ＜0.001 |  |  |  | < 0.001 |  |  |  | < 0.001 |
| Never smoker | 1266 (68.66) | 446 (71.28) | 432 (69.75) | 388 (64.45) |  | 440 (61.13) | 441(72.12) | 385 (70.70) |  | 321 (60.28) | 319 (69.96) | 626 (70.53) |  |
| Former smoker | 185 (12.96) | 76 (17.44) | 61 (11.37) | 48 (9.74) |  | 58 (11.97) | 58 (11.70) | 69 (16.64) |  | 40 (10.95) | 60 (17.89) | 85 (12.17) |  |
| Current smoker | 323 (18.38) | 70 (11.28) | 98 (18.88) | 155 (25.81) |  | 164 (26.90) | 95 (16.18) | 64 (12.66) |  | 133 (28.77) | 51 (12.15) | 139 (17.30) |  |
| **Physical activity (%)** |  |  |  |  | 0.17 |  |  |  | < 0.001 |  |  |  | < 0.001 |
| ≥ 600 MET-min/week^b^ | 1161 (70.19) | 410 (73.91) | 382 (69.98) | 369 (66.24) |  | 404 (63.83) | 389 (69.94) | 368 (77.70) |  | 305 (62.91) | 308 (78.64) | 548 (69.47) |  |
| < 600 MET-min/week | 613 (29.81) | 182 (26.09) | 209 (30.02) | 222 (33.76) |  | 258 (36.17) | 205 (30.06) | 150 (22.30) |  | 189 (37.09) | 122 (21.36) | 302 (30.53) |  |
| **Total energy intake, kcal** | 1904.40 (23.087) | 2332.33 (38.847) | 1910.10 (27.421) | 1438.73 (29.555) | < 0.001 | 1426.87 (22.735) | 1916.18 (27.242) | 2476.04 (46.381) | < 0.001 | 1340.13 (28.367) | 2500.14 (52.194) | 1904.93 (24.062) | < 0.001 |
| **BMI^b^, kg/m^2^** | 29.22 (0.25) | 27.95 (0.43) | 29.49 (0.44) | 30.30 (0.34) | < 0.001 | 30.25 (0.34) | 28.58 (0.39) | 28.71 (0.53) | 0.016 | 30.64 (0.45) | 28.38 (0.49） | 28.90 (0.36） | 0.002 |
| **Obese^c^ (%)** |  |  |  |  | 0.08 |  |  |  | 0.023 |  |  |  | 0.025 |
| No | 1046 (61.38) | 382 (67.15) | 351 (59.57) | 313 (56.94) |  | 351 (55.35) | 381 (65.20) | 314 (63.97) |  | 251 (53.13) | 267 (65.20) | 528 (63.61) |  |
| Yes | 728 (38.62) | 210 (32.85) | 240 (40.43) | 278 (43.06) |  | 311 (44.65) | 213 (34.80) | 204 (36.03) |  | 243 (46.87) | 163 (34.80) | 322 (36.39) |  |
| **WC^b^, cm** | 95.54 (0.60) | 92.91 (1.00) | 95.87 (0.85) | 98.01 (0.88) | < 0.001 | 97.73 (0.87) | 94.41 (0.89) | 94.18 (1.24) | 0.036 | 98.70 (1.18) | 93.47 (1.22) | 94.91 (0.75) | 0.004 |
| **High-WC^c^ (%)** |  |  |  |  | 0.008 |  |  |  | 0.015 |  |  |  | 0.008 |
| No | 671 (40.04) | 255 (45.13) | 230 (40.52) | 186 (34.06) |  | 211 (34.46) | 252 (43.43) | 208 (42.93) |  | 146 (32.40) | 181 (45.04) | 344 (41.57) |  |
| Yes | 1103 (59.96) | 337 (54.87) | 361 (59.48) | 405 (65.94) |  | 451 (65.54) | 342 (56.57) | 310 (57.07) |  | 348 (67.60) | 249 (54.96) | 506 (58.43) |  |
| **Infertility (%)** |  |  |  |  | 0.03 |  |  |  | 0.19 |  |  |  | 0.026 |
| No | 1561 (87.34) | 531 (91.32) | 519 (86.33) | 511 (83.61) |  | 581 (87.27) | 517 (85.51) | 463 (89.58) |  | 430 (85.33) | 388 (90.76) | 743 (86.71) |  |
| Yes | 213 (12.66) | 61 (8.68) | 72 (13.67) | 80 (16.39) |  | 81(12.73) | 77 (14.49) | 55 (10.42) |  | 64 (14.67) | 42 (9.24) | 107 (13.29) |  |

^a^ Values are presented as weighted mean (SE) for continuous variables, and n (%) for categorical variables.

^b^ T, tertile; PIR, family income-to-poverty ratio; MET, metabolic equivalent; BMI, body mass index; WC, waist circumference.

^c^ Individuals with a BMI≥30.0 kg/m^2^ were classified as “obese”, and women with a WC≥88 cm were labeled as “high-WC” (22).

^d^ ANOVA was used for differences in weighted means of continuous variables, and the chi-squared test was used for differences in weighted percentages of categorical variables.

**Supplementary Table 4.** Associations of DII, DOBS, and DII&DOBS with indicators of reproductive function in binomial logistic regression models (n=767). ^a^

| **Subgroup** | **Infertility** | **SHBG**^b^ | **TT**^b^ | **E_2_**^b^ |
| --- | --- | --- | --- | --- |
| **DII**^b^ | 1.17 (0.98-1.40) | 0.95 (0.85-1.07) | 1.05 (0.94-1.18) | 1.09 (0.98-1.22) |
| T1 | Ref. | Ref. | Ref. | Ref. |
| T2 | 1.45 (0.78-2.70) | 0.90 (0.60-1.38) | 0.92 (0.62-1.37) | 1.13 (0.77-1.66) |
| T3 | 1.59 (0.82-3.16) | 0.88 (0.55-1.40) | 1.16 (0.74-1.83) | 1.12 (0.73-1.72) |
| **DOBS**^b^ | 0.97 (0.93-1.02) | 1.01 (0.98-1.05) | 0.99 (0.96-1.03) | 0.98 (0.95-1.01) |
| T1 | Ref. | Ref. | Ref. | Ref. |
| T2 | 1.06 (0.60-1.87) | 0.89 (0.59-1.33) | 1.09 (0.74-1.63) | 1.09 (0.75-1.59) |
| T3 | 0.75 (0.36-1.51) | 1.22 (0.75-2.00) | 1.04 (0.64-1.67) | 0.82 (0.52-1.30) |
| **DII&DOBS** |  |  |  |  |
| Pro-inflammatory and pro-oxidative diet | Ref. | Ref. | Ref. | Ref. |
| Anti-inflammatory and anti-oxidative diet | 0.43 (0.18-0.95)^*^ | 1.25 (0.72-2.17) | 0.86 (0.51-1.47) | 0.69 (0.41-1.14) |
| Composite diet | 0.75 (0.43-1.30) | 0.99 (0.66-1.49) | 0.86 (0.58-1.28) | 0.96 (0.66-1.40) |

^a^ Values are presented as OR and 95% confidence intervals. SHBG, TT, and E_2_ were transformed into binary variables by comparing them to their respective medians. Models were adjusted for age, race, education, marital status, PIR, smoking status, physical activity, total energy intake, total cholesterol, triglyceride, LDL-cholesterol, HDL-cholesterol and HOMA-IR. ^*^*p*<0.05.

^b^ DII, dietary inflammatory index; DOBS, dietary oxidative balance score; SHBG, sex hormone-binding globulin; TT, total testosterone; E_2_, estradiol; PIR, family income-to-poverty ratio; HOMA-IR, insulin resistance index.


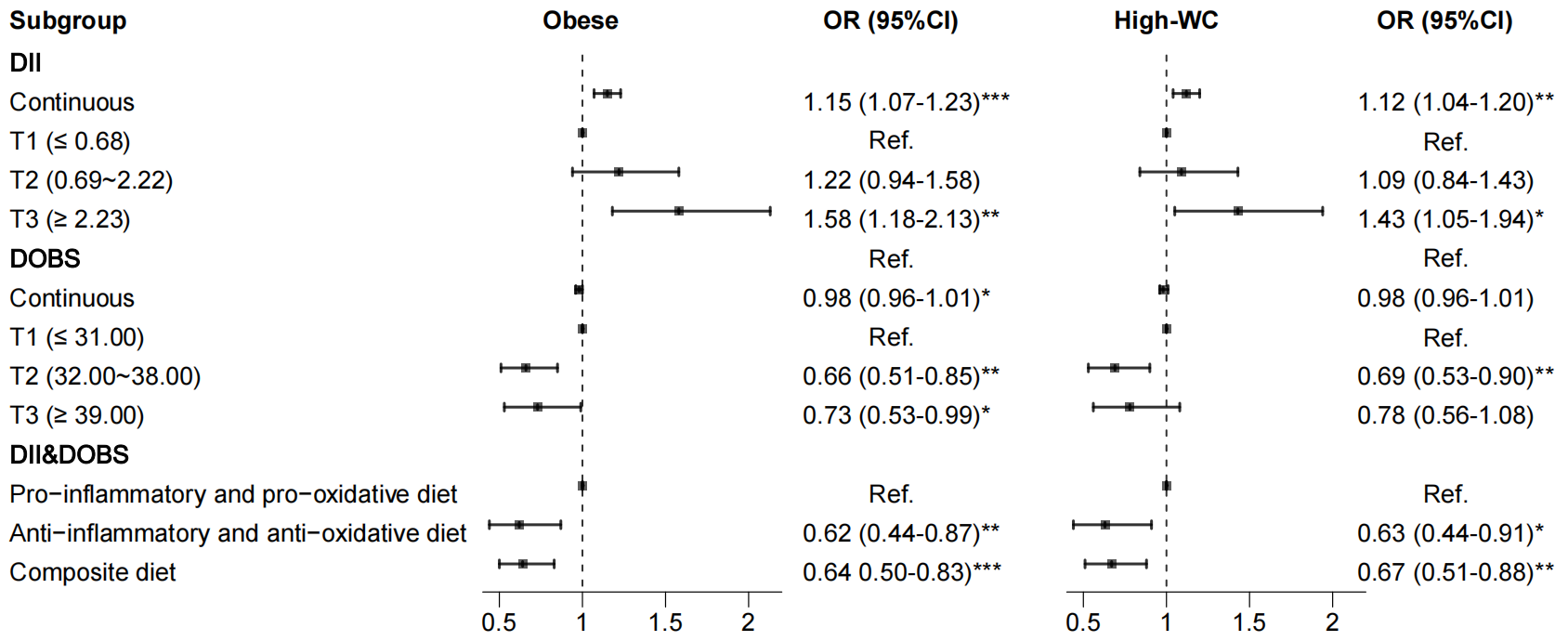


**Supplementary Figure 1.** Forest plot of the associations of DII, DOBS, and DII&DOBS with obesity indicators in binomial logistic regression models. Models were adjusted for age, race, education, marital status, PIR, smoking status, physical activity and total energy intake. ^*^*p*<0.05, ^**^*p*<0.01, ^***^*p*<0.001. DII, dietary inflammatory index; DOBS, dietary oxidative balance score; T, tertile; WC, waist circumference; PIR, family income-to-poverty ratio.


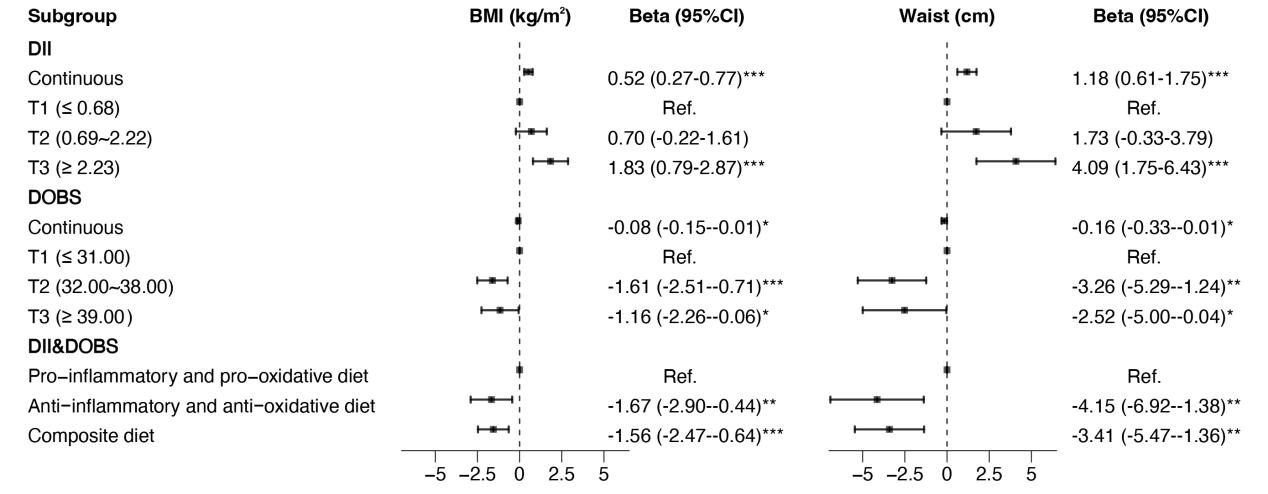


**Supplementary Figure 2.** Forest plot of the associations of DII, DOBS, and DII&DOBS with obesity indicators in multivariate linear regression models. Models were adjusted for age, race, education, marital status, PIR, smoking status, physical activity and total energy intake. ^*^*p*<0.05, ^**^*p*<0.01, ^***^*p*<0.001. DII, dietary inflammatory index; DOBS, dietary oxidative balance score; T, tertile; BMI, body mass index; PIR, family income-to-poverty ratio.


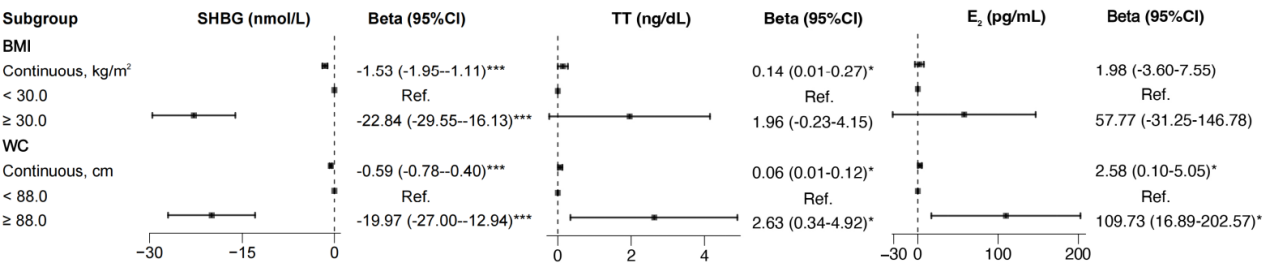


**Supplementary Figure 3.** Forest plot of the associations of BMI, WC with SHBG and sex hormones in multivariate linear regression models. Models were adjusted for age, race, education, marital status, PIR, smoking status, physical activity and total energy intake. ^*^*p*<0.05, ^***^*p*<0.001. SHBG, sex hormone-binding globulin; TT, total testosterone; E_2_, estradiol; BMI, body mass index; WC, waist circumference; PIR, family income-to-poverty ratio.


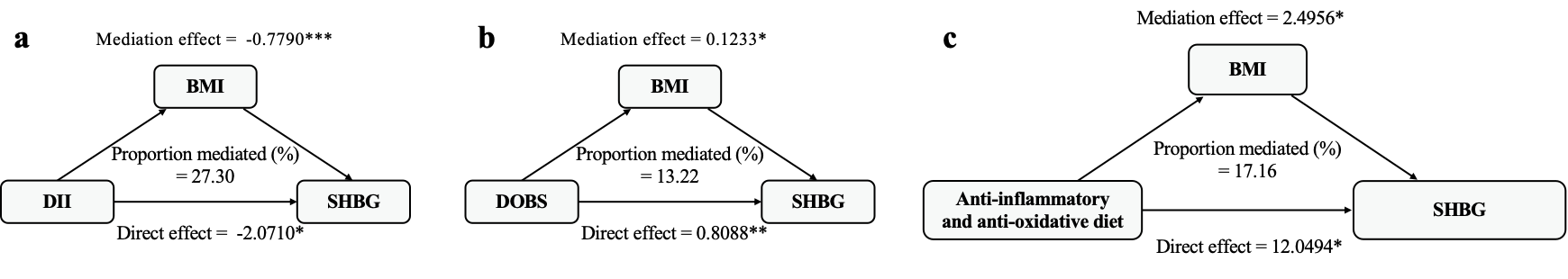


**Supplementary Figure 4.** BMI mediated the relationships between DII (a), DOBS (b), anti-inflammatory and anti-oxidative diet (c) and SHBG. Models were adjusted for age, race, education, marital status, PIR, smoking status, physical activity, total energy intake. ^*^*p*<0.05, ^**^*p*<0.01, ^***^*p*<0.001. DII, dietary inflammatory index; DOBS, dietary oxidative balance score; BMI, body mass index; SHBG, sex hormone-binding globulin; PIR, family income-to-poverty ratio.


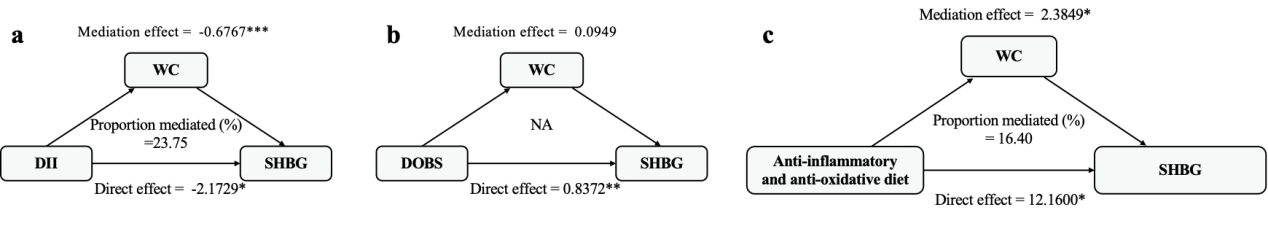


**Supplementary Figure 5.** WC mediated the relationships between DII (a), anti-inflammatory and anti-oxidative diet (c) and SHBG. Models were adjusted for age, race, education, marital status, PIR, smoking status, physical activity, total energy intake. ^*^*p*<0.05, ^**^*p*<0.01, ^***^*p*<0.001. DII, dietary inflammatory index; DOBS, dietary oxidative balance score; WC, waist circumference; SHBG, sex hormone-binding globulin; PIR, family income-to-poverty ratio; NA, not applicable.
